# Changing causes of death in persons with haematological cancers 1975–2016

**DOI:** 10.1038/s41375-022-01596-z

**Published:** 2022-05-16

**Authors:** Lezong Chen, Yongqiang Zheng, Kai Yu, Shuzhao Chen, Weida Wang, Robert Peter Gale, Ze-Xian Liu, Yang Liang

**Affiliations:** 1 State Key Laboratory of Oncology in South China; Collaborative Innovation Center for Cancer Medicine, Sun Yat-sen University Cancer Center, Guangzhou, 510060 Guangdong Province China; 2 Haematology Research Centre, Department of Immunology and Inflammation, Imperial College London, London, UK

**Keywords:** Haematological diseases, Epidemiology, Prognosis, Public health

## Abstract

Causes of death in persons with haematological cancers include the index cancer, a new cancer or a seemingly unrelated cause such as cardio-vascular disease. These causes are complex and sometimes confounded. We analyzed trends in cause of death in 683,333 persons with an index haematological cancer diagnosed in 1975–2016 reported in the Surveillance, Epidemiology and End Results dataset. Non-cancer deaths were described using standardized mortality ratios. The index cancer was the predominant cause of death amongst persons with plasma cell myeloma, acute lymphoblastic leukaemia and acute myeloid leukaemia. Non-cancer death was the major cause of death in persons with chronic lymphocytic leukaemia, Hodgkin lymphoma and chronic myeloid leukaemia, mostly from cardio-vascular diseases. The greatest relative decrease in index-cancer deaths was amongst persons with Hodgkin lymphoma, chronic myeloid leukaemia and chronic lymphocytic leukaemia, where the proportion of non-cancer deaths increased substantially. Changing distribution of causes of death across haematological cancers reflects substantial progress in some cancers and suggests strategies to improve the survival of persons with haematological cancers in the future.

## Introduction

Haematological cancers are an important cause of death worldwide [1]. In 2020, there were an estimated 1.3 million new haematological cancers with 700,000 deaths worldwide. Incidences of most haematological cancers increase with age. The Global Burden of Disease (GBD) study reported that incidences of non-Hodgkin lymphoma (NHL) and leukaemias increased 45 percent and 26 percent from 2006 to 2016 [2].

There are substantial recent advances in therapy of some haematological cancers such as Hodgkin lymphoma (HL) and NHL, chronic myeloid and lymphocytic leukaemias (CML and CLL) and plasma cell myeloma (PCM) [3–7]. There are more modest recent advances in other haematological cancers such as acute myeloid and lymphoid leukaemias (AML and ALL). Much of this progress has resulted in increased survival rather than increased cures [8]. Survivors of cancer are at risk for a range of late-occurring side effects from treatment, which would also change the fate of these patients [9]. In older persons with a haematological cancer, increased survival after a cancer diagnosis may result in more deaths from age-related diseases such as cardio-vascular disease (CVD), stroke and diabetes.

Analyses of causes of death in persons with a haematological cancer are complex. For example, as deaths from some cancers such as HL have decreased the proportion of deaths from new cancers, such as therapy-related AML and CVD, have increased [10, 11]. In CML, where survival has increased substantially, the proportion of deaths from non-CML causes has increased markedly [12]. Sometimes there is uncertainty to identify the cause(s) of death in persons with a haematologic cancer. For example, some persons with AML receiving a haematopoietic cell transplant whilst in 1^st^ remission die from transplant-related mortality, such as graft-*versus*-host disease (G*v*HD) [13]. If these persons were cured before they received a transplant, it is unknown whether the death should be scored as from AML, the transplant or G*v*HD.

We interrogated data from the Surveillance, Epidemiology and End Results (SEER) dataset 1975–2016 to assess the trends for cancer-related and 26 major types of non-cancer cause(s) of death of persons with a haematological cancer.

## Subjects and methods

### Data source and study population

We performed a population-based cohort study using data from the Surveillance, Epidemiology and End Results (SEER) programme, a population-based cancer registry with data on cancer demographics, incidence, anatomic site, histology, stage, therapy, socio-economic state and vital statistics of about one-third of persons with cancer in the US [14].

We extracted data on all first primary newly-diagnosed haematologic cancers diagnosed between 1975 and 2016 from the SEER 18 database (2019 Edition) using SEER*Stat software version 8.3.8 [15]. Diagnosis was based on coding in International Classification of Diseases for Oncology 3rd edition [ICD-O-3] codes, 9590–9992. Subjects were excluded if their diagnoses were obtained only from death certificates or autopsies. We also excluded subjects without active follow-up and those with unknown follow-up time, age at diagnosis and/or cause of death (Fig. S1). To analyze the causes of death as a function of calendar year of death, we extracted cause of death data amongst persons with a first haematological cancer diagnosis between 1975 and 2016 using the SEER-9 incidence-based mortality session [16]. For comparison with the general population, we extracted sex-, age-, race- and year-stratified mortality data of the US general population between 1975 and 2016 from the National Center for Health Statistics [17].

The Sun Yat-sen University Cancer Center (SYSUCC) Institutional Review Board (IRB) waived the requirement for a Research Data Agreement and informed consent consistent with principles of the Declaration of Helsinki.

### Co-variates analyzed

Follow-up from diagnosis was defined as the interval between cancer diagnosis and death from any cause, last follow-up or the end of the study on December 31, 2016. Interrogated co-variates included sex, race, age at diagnosis, year of cancer diagnosis, cancer histology, follow-up duration and cause of death.

We classified haematological cancers by histology including: (1) HL (ICD-O-3 codes: 9650-9667); (2) NHL (ICD-O-3 codes: 9590-9597, 9670-9671, 9673, 9675, 9678-9680, 9684, 9687-9691, 9695, 9698-9702, 9705, 9708-9709, 9712, 9714-9719, 9724-9729, 9735, 9737-9738, 9811-9818, 9823, 9827, 9837); (3) PCM (ICD-O-3 codes: 9731-9732, 9734); (4) ALL (ICD-O-3 codes: 9811-9818, 9826, 9835-9837); (5) CLL (ICD-O-3 codes: 9823); (6) AML (ICD-O-3 codes: 9840, 9861, 9865-9867, 9869, 9871-9874, 9891, 9895-9897, 9898, 9910-9911, 9920); (7) CML (ICD-O-3 codes: 9863, 9875-9876, 9945-9946: and (8) other leukaemias (ICD-O-3 codes: 9733, 9742, 9800-9801, 9805-9809, 9820, 9831-9834, 9860, 9870, 9930-9931, 9940, 9948, 9963-9964).

Cause of death was defined using the International Statistical Classification of Diseases and Related Health Problems [ICD]. Because death codes changed over the interval we studied, we used ICD-8 codes for cases diagnosed from 1975 to 1978, ICD-9 codes for cases diagnosed from 1979 to 1998 and ICD-10 codes for cases diagnosed from 1999 to 2016. The SEER programme summarized these codes and generated a combined death code.

We classified causes of death into three groups: (1) index-cancer; (2) non-index-cancer; or (3) non-cancer. Causes of death were defined by SEER cause-specific death classification variable from death certificates [18, 19]. Non-cancer causes were categorized into 26 major groups which were consolidated into 7 broad categories: (1) infection; (2) CVD; (3) respiratory disease; (4) gastro-intestinal disease; (5) kidney disease; (6) external injuries; and (7) other. Deaths from in situ, benign or unknown behavior neoplasms are classified as non-cancer deaths by SEER programme [18, 19]. We did not include these deaths in the non-cancer deaths. These deaths also cannot be classified into cancer-related deaths; thus, they were excluded from our analyses (3,534 cases were excluded [0.5%]; Fig. S1). In the SEER programme, survival is measured in months. Subjects with survival < 1 month were recorded as 0 months according to standard epidemiologic convention [20].

### Statistical analyses

Mortality rates were calculated as number of deaths divided by the person-years of follow-up. For non-cancer deaths, standardized mortality ratios (SMRs) and 95% Confidence Intervals (CIs) were calculated for comparison with the US population [19, 21]. SMR was estimated as ratio of observed to expected number of deaths. Observed number of deaths represents the number of deaths from certain causes in persons with cancer and expected number of deaths represents numbers of people dying from the same causes in the general population with a similar distribution of age, sex, race and calendar year. All-cause and cause-specific SMRs were calculated among patients with haematological cancer.

To obtain the expected number of deaths, we derived the stratum-specific mortality rates from the same cause in the reference general population collected by the SEER programme and calculated person-years of relevant strata in the haematological cancer cohort. The stratum-specific expected number of deaths was estimated as the product of death rate in the reference cohort and person-years in the haematological cancer cohort. Total expected number of deaths is the sum of all expected deaths across the strata.

Age at diagnosis was divided into 19 cohorts: (1) 00 years; (2) 01–04 years; (3) 05–09 years; (4) 10–14 years; (5) 15–19 years; (6) 20–24 years; (7) 25–29 years; (8) 30–34 years; (9) 35–39 years; (10) 40–44 years; (11) 45–49 years; (12) 50–54 years; (13) 55–59 years; (14) 60–64 years; (15) 65–69 years; (16) 70–74 years; (17) 75–79 years; (18) 80–84 years; and (19) 85+ years. Year of haematological cancer diagnosis was divided into 8 cohorts: (1) 1975–1979; (2) 1980–1984; (3) 1985–1989; (4) 1990–1994; (5) 1995–1999; (6) 2000–2004; (7) 2005–2009; and (8) 2010–2016. 95% confidence intervals (CIs) of SMRs were obtained using an approximation from a Poisson distribution [20, 22]. The Kaplan-Meier method was used to estimate actuarial probability of death amongst persons with a haematological cancer and competing risk analyses were done to estimate risk of dying from the index cancer, non-index cancer and non-cancer causes.

Data on deaths were extracted from SEER 9 registries databases which continually code death trends from diverse causes by calendar year of cancer diagnosis from 1975 to 2016. In the trend analyses, we restricted follow-up interval to avoid bias from different intervals of follow-up time for persons diagnosed in different periods. For example, subjects diagnosed 1975–1979 were followed to 1984 (an additional 5-year minimum duration), those diagnosed 1980–1984 were followed to 1989, and those diagnosed in 2010–2014 were followed to 2016. All deaths during follow-up were recorded. Cancers diagnosed 2015–2016 were excluded because of inadequate follow-up. Joinpoint Software was used to fit the trends of deaths from index cancer, non-index cancer and non-cancer causes [23]. Joinpoint Software generated one or more curves based on a log-linear regression model and so-called *joinpoints*, which were used to connect adjacent curves to generate a continuous curve spanning the study interval. Percentage change per 5 years for each trend was estimated and integrated to generate an average 5-year percentage change for the entire trend. We describe risk of death from non-cancer causes as a function of haematological cancer-type and interval from haematological cancer diagnosis.

Tests were two-sided and *P*-values < 0.05 were considered statistically significant. Analyses were done with SEER*Stat software version 8.3.6, the R version 3.63 statistical software, and Joinpoint Regression Software version 4.7.0.0 [15, 23, 24].

## Results

683,333 persons with a haematological cancer diagnosed from 1975 to 2016 were included (Fig. S1). Median follow-up from index cancer diagnosis was 3 years (range, 0 to 42 years). 55 percent were male and 83 percent were white. Median age at diagnosis was 64 years (Inter-Quartile Range [IQR], 0–109 years). 46 percent had an NHL, 15 percent, PCM, 11 percent, CLL, 9 percent, AML, 5 percent, ALL, and 4 percent, CML. Baseline co-variates are displayed in Table 1.

**Table 1:** Baseline co-variates of patients with haematologic cancers, 1975-2016.

Baseline co-variates	*N* (%)	Total Follow-up time (years)	Deaths from all causes (%)	Deaths from non-cancer causes	
				No. of observed deaths (%)	SMR (95% CI)
All	683,333 (100%)	3,755,161	390,534 (100%)	113,172 (100%)	2.51 (2.49–2.52)
Age					
0–19	39,847 (6%)	404,726	8616 (2%)	1280 (1%)	5.96 (5.64–6.30)
20–39	70,378 (10%)	634,977	23,146 (6%)	6998 (6%)	9.31 (9.09–9.53)
40–59	175,767 (26%)	1,182,981	78,393 (20%)	19,885 (18%)	4.45 (4.39–4.51)
60–79	292,883 (43%)	1,306,862	191,340 (49%)	54,651 (48%)	2.55 (2.53–2.57)
80+	104,458 (15%)	225,615	89,039 (23%)	30,358 (27%)	1.66 (1.64–1.68)
Sex					
Female	305,627 (45%)	1,722,851	172,502 (44%)	47,833 (42%)	2.44 (2.42–2.46)
Male	377,706 (55%)	2,032,311	218,032 (56%)	65,339 (58%)	2.55 (2.53–2.57)
Race					
White	567,502 (83%)	3,187,711	329,103 (84%)	95,587 (85%)	2.40 (2.39–2.42)
Black	66,016 (10%)	315,522	38,769 (10%)	12,166 (11%)	3.21 (3.15–3.26)
Other	49,815 (7%)	251,928	22,662 (6%)	5,419 (5%)	3.41 (3.32–3.50)
Year of diagnosis					
1975–1989	89,574 (13%)	708,262	80,674 (21%)	23,170 (21%)	3.08 (3.05–3.12)
1990–1999	107,421 (16%)	826,692	84,143 (22%)	26,284 (23%)	2.98 (2.95–3.02)
2000–2009	271,978 (40%)	1,707,398	159,751 (41%)	47,098 (42%)	2.21 (2.19–2.23)
2010–2016	214,360 (31%)	512,810	65,966 (17%)	16,620 (15%)	2.21 (2.18–2.24)
Marital states					
Married	353,507 (52%)	2,006,942	203,867 (52%)	54,232 (48%)	2.25 (2.23–2.27)
Umarried	282,973 (41%)	1,496,235	165,341 (42%)	51,172 (45%)	2.96 (2.94–2.99)
Unknown	46,853 (7%)	251,984	21,326 (5%)	7768 (7%)	2.05 (2.01–2.10)
Radiotherapy					
Yes	111,543 (16%)	845,788	58,543 (15%)	16,751 (15%)	2.45 (2.42–2.49)
No/Unknown	575,324 (84%)	2,920,824	335,525 (86%)	96,421 (85%)	2.51 (2.50–2.53)
Chemotherapy					
Yes	397,829 (58%)	2,232,639	220,212(56%)	50,599 (45%)	2.49 (2.47–2.51)
No/Unknown	285,504 (42%)	1,522,522	170,322(44%)	62,573 (55%)	2.51 (2.50–2.53)
Diagnosis					
ALL	30,884 (5%)	234,548	11,505 (3%)	1283 (1%)	5.25 (4.97–5.55)
AML	60,065 (9%)	143,407	47,324 (12%)	4745 (4%)	5.13 (4.99–5.28)
CLL	76,594 (11%)	472,786	42,383 (11%)	18,636 (17%)	1.87 (1.84–1.90)
CML	28,985 (4%)	133,082	16,744 (4%)	5054 (5%)	3.27 (3.18–3.36)
HL	53,589 (8%)	546,752	16,413 (4%)	6183 (6%)	3.81 (3.71–3.90)
PCM	101,140 (15%)	349,177	71,692 (18%)	17,280 (15%)	2.79 (2.75–2.83)
NHL	311,250 (46%)	1,780,946	170,080 (44%)	56,270 (50%)	2.40 (2.38–2.42)
Other leukemias	20,826 (3%)	94,463	14,393 (4%)	3721 (3%)	3.05 (2.96–3.15)

### Causes of death by calendar interval

**Fig. 1:**
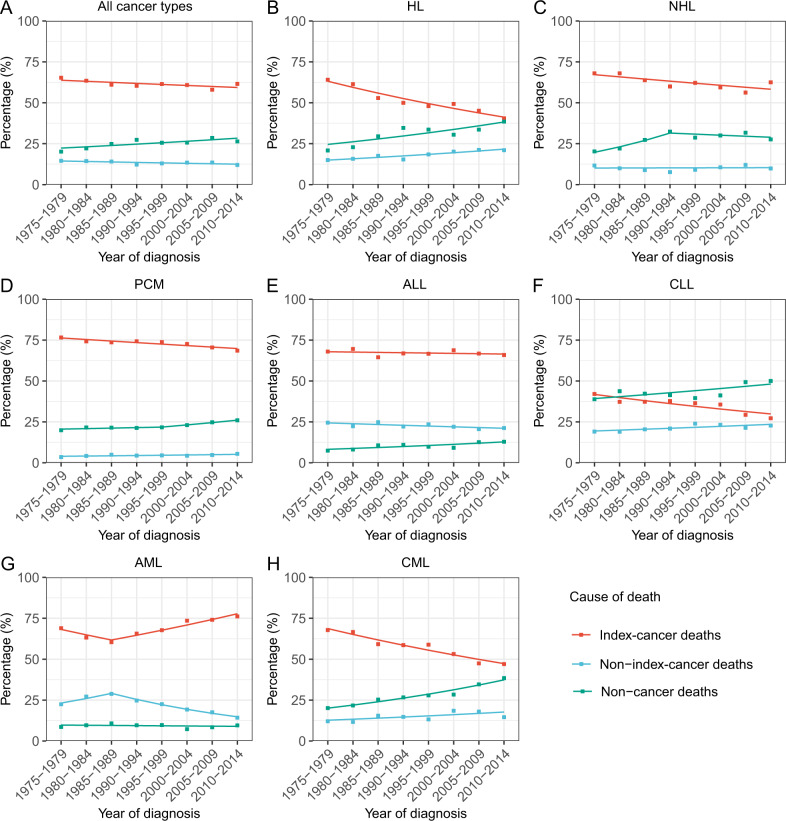
Deaths from the index cancer, non-index cancer or non-cancer causes among patients with haematological cancer by year of diagnosis from 1975 to 2016. **A** All haematological cancers, **B** HL, **C** NHL, **D** PCM, **E** ALL, **F** CLL, **G** AML, **H** CML.

We analyzed death trends by assessing deaths from index cancer, other cancer or non-cancer causes by cancer type (Fig. 1). In persons diagnosed in 1975–1979, index-cancer deaths accounted for 65 percent of deaths, 2^nd^ cancer for 15 percent and non-cancer deaths, 20 percent. In persons diagnosed in 2010–2014, index-cancer deaths decreased to 62 percent (*p* = 0.04), 2^nd^ cancers decreased to 12 percent (*p* = 0.04) and non-cancer deaths increased to 26 percent (*p* = 0.02).

Persons most likely to die from their index cancer were those with PCM, ALL and AML. The proportion of deaths from index cancer remained stable or changed slightly for ALL (*p* = 0.5). For AML, there was a relative increase in index-cancer deaths beginning in 1985 (1975–1979 to 1985–1989: *p* = 0.2; 1985–1989 to 2010–2014: *p* = 0.01), with an insignificant overall trend (*p* = 0.07). In contrast, there was a marked decrease in index-cancer deaths in persons with HL, CML, CLL (HL: *p* < 0.001; CML: *p* < 0.001; CLL: *p* = 0.004) and a modest decrease in index-cancer deaths in persons with NHL and PCM (NHL: *p* = 0.02; PCM: *p* = 0.001;) (Fig. 1 and Table S1).

For HL, CML and CLL, non-cancer deaths increased continually 1975–2014 (HL: *p* = 0.01; CML: p < 0.001; CLL: *p* = 0.04). For PCM and ALL, although non-cancer deaths were less frequent, there was an increased trend (PCM: *p* = 0.001; ALL: *p* = 0.01). For NHL, non-cancer deaths increased substantially 1975-1994, and remaind stable thereafter (1975–1979 to 1990–1994: *p* = 0.07; 1990–1994 to 2010–2014: *p* = 0.5). Beginning in 1980s, non-cancer causes became the most common causes of death in persons with CLL (Fig. 1 and Table S1).

Non-index-cancer deaths increased in persons with HL, CLL (HL: PC = 5.41; *p* = 0.001; CLL: *p* = 0.03), but decreased in ALL and AML (ALL: *p* = 0.03; AML: *p* < 0.001) (Fig. 1 and Table S1).

### Causes of death by cancer type

**Fig. 2:**
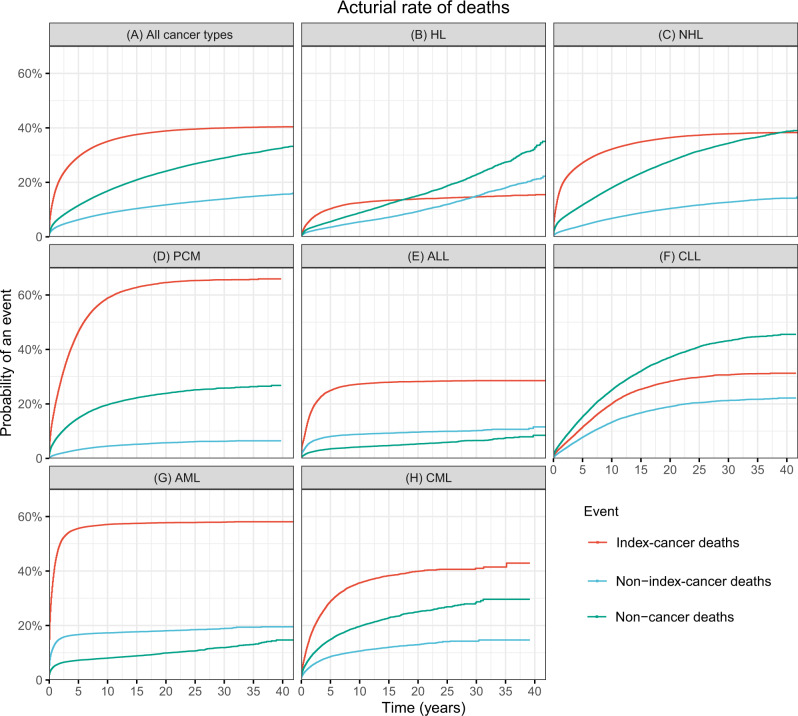
Actuarial rates of deaths from index cancer, a new cancer or a non-cancer cause based on competing risks. **A** All haematological cancers, **B** HL, **C** NHL, **D** PCM, **E** ALL, **F** CLL, **G** AML, **H** CML.

There were 390,534 deaths amongst 683,333 persons with a haematological cancer (57%) diagnosed 1975–2016. 56 percent of deaths were from the index cancer (*N* = 219,731), 15 percent from a 2^nd^ cancer (*N* = 57,631) and 29 percent (*N* = 113,172) from non-cancer causes, predominately CVD (13%, *N* = 49,498) (Table 2). The 10-year actuarial rate of all-cause deaths was 57.8% (57.6%, 57.9%). 10-year actuarial rates were 35.0% (34.9%, 35.2%) for index-cancer deaths, 8.7% (8.6%, 8.7%) for non-index-cancer deaths, and 16.9% (16.7%, 17.0%) for non-cancer deaths (Fig. 2).

**Table 2:** Causes of deaths among patients with haematologic cancers.

Causes of death	Cancer		General population		SMR (95% CI)
	Observed No. of deaths (%)	Mortality rates in cancer patients	Expected No. of deaths	Mortality rates in general population	
**All causes**	**390,534 (100%)**	**10,399.9**	NA	NA	NA
**Index cancer**	**219,731 (56%)**	**5833.7**	NA	NA	NA
**Non-index cancer**	**57,631 (15%)**	**1534.7**	NA	NA	NA
**Non-cancer causes**	**113,172 (29%)**	**3004.6**	**45176.4**	**1199.4**	**2.51 (2.49–2.52)**
**Infections**	**19,111 (5%)**	**507.4**	**2933.4**	**77.9**	**6.51 (6.42–6.61)**
Pneumonia and influenza	5748 (2%)	152.6	1609.8	42.7	3.57 (3.48–3.66)
Syphilis	1 (0.0003%)	0.03	0.8	0.02	1.19 (0.17–8.44)
Tuberculosis	73 (0.02%)	1.9	29.8	0.8	2.45 (1.95–3.08)
Septicemia	2489 (1%)	66.1	719.0	19.1	3.46 (3.33–3.60)
Other infections	10,800 (3%)	286.7	572.9	15.2	18.9 (18.5–19.2)
**Cardio-vascular diseases**	**49,498 (13%)**	**1314.1**	**23825.7**	**632.5**	**2.08 (2.06–2.10)**
Diseases of heart	39,515 (10%)	1049.1	18469.6	490.3	2.14 (2.12–2.16)
Hypertension without heart disease	1353 (0.3%)	35.9	467.4	12.4	2.89 (2.74–3.05)
Aortic aneurysm and dissection	545 (0.1%)	14.5	420.3	11.2	1.30 (1.19–1.41)
Atherosclerosis	710 (0.2%)	18.8	347.6	9.2	2.04 (1.90–2.20)
Cerebrovascular diseases	6855 (2%)	182.0	3871.4	102.8	1.77 (1.73–1.81)
Other diseases of arteries, arterioles, capillaries	520 (0.1%)	13.8	248.6	6.6	2.09 (1.92–2.28)
**Respiratory diseases**	**6405 (2%)**	**170.0**	**3374.4**	**89.6**	**1.90 (1.85–1.95)**
Chronic obstructive pulmonary disease and allied cond	6405 (2%)	170.0	3374.4	89.6	1.90 (1.85–1.95)
**Gastrointestinal diseases**	**1746 (0.4%)**	**46.4**	**866.6**	**23.0**	**2.01 (1.92–2.11)**
Stomach and duodenal ulcers	366 (0.1%)	9.7	122.5	3.3	2.99 (2.70–3.31)
Chronic liver disease and cirrhosis	1380 (0.4%)	36.6	743.9	19.7	1.86 (1.76–1.96)
**Renal diseases**	**3234 (0.8%)**	**85.9**	**935.0**	**24.8**	**3.46 (3.34–3.58)**
Nephritis, nephrotic syndrome and nephrosis	3234 (0.8%)	85.9	935.0	24.8	3.46 (3.34–3.58)
**External injuries**	**4717 (1%)**	**125.2**	**2725.3**	**72.4**	**1.73 (1.68–1.78)**
Accidents and adverse effects	3620 (0.9%)	96.1	1923.7	51.1	1.88 (1.82–1.94)
Suicide and self-inflicted injury	958 (0.2%)	25.4	586.5	15.6	1.63 (1.53–1.74)
**Other cause of death**	**28,461 (7%)**	**755.6**	**10515.7**	**279.2**	**2.71 (2.68–2.74)**
Homicide and legal intervention	139 (0.04%)	3.7	214.9	5.7	0.65 (0.55–0.76)
Alzheimer’s diseases	2337 (0.6%)	62.0	1191.0	31.6	1.96 (1.88–2.04)
Diabetes mellitus	3115 (0.8%)	82.7	1734.9	46.1	1.80 (1.73–1.86)
Congenital anomalies	313 (0.1%)	8.3	95.5	2.5	3.28 (2.93–3.66)
Certain conditions originating in perinatal period	16 (0.004%)	0.4	29.5	0.8	0.54 (0.33–0.88)
Complications of pregnancy, childbirth, puerperium	54 (0.01%)	1.4	3.0	0.1	17.7 (13.6–23.2)
Symptoms, signs and ill-defined conditions	1498 (0.4%)	39.8	614.8	16.3	2.44 (2.32–2.56)
Other cause of death	21,128 (5%)	560.9	6845.6	181.7	3.09 (3.05–3.13)

The bold values are the indicate major groups.

The all-cause SMR for non-cancer deaths was 2.51 (2.49, 2.52) compared with the general population. Compared with cause-specific mortality in the general population, risks were highest for infections and kidney diseases, with cause-specific SMRs of 6.51 (6.42, 6.61) and 3.46 (3.34, 3.58), respectively. There were also increased risks of death for pregnancy, childbirth and puerperium complications (cause-specific SMR = 17.7 [13.6, 23.2]) (Table 2).

### Index-cancer deaths

**Fig. 3:**
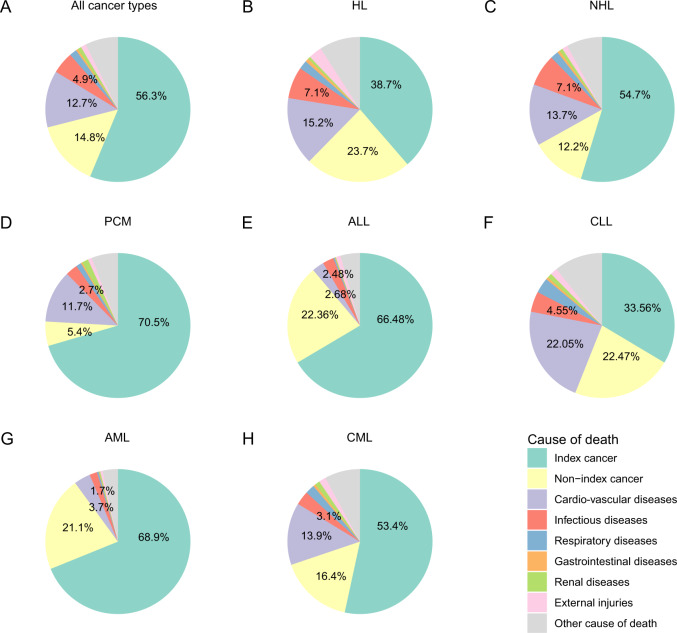
Distribution of causes of death. **A** All haematological cancers, **B** HL, **C** NHL, **D** PCM, **E** ALL, **F** CLL, **G** AML, **H** CML.

The index cancer accounted for a large proportion of deaths in persons with i PCM (70%), AML (68%), ALL (66%), NHL (54%), CML (52%), and for a smaller proportion of deaths in persons with HL (39%) and CLL (33%) (Fig. 3; Table S1 to S7). 10-year actuarial rates of index-cancer deaths were 58.9% (58.5%, 59.2%) for PCM, 57.1% (56.7%, 57.5%) for AML, 35.7% (35.0%, 36.3%) for CML, 32.2% (32.0%, 32.4%) for NHL, 27.3% (26.8%, 27.8%) for ALL, 20.1% (19.7%, 20.4%) for CLL and 12.5% (12.2%, 12.8%) for HL (Fig. 2).

### Non-index cancer deaths

Non-index cancer accounted for a large proportion of deaths in persons with HL (24%), CLL (23%), ALL (23%) and AML (22%) and for a smaller proportion of deaths in persons with NHL (13%) and PCM (6%; Fig. 3, Tables S2–S8). 10-year actuarial rates of non-index-cancer deaths were 17.3% (17.0%, 17.6%) for AML, 13.3% (13.0%, 13.6%) for CLL, 10.6% (10.2%, 11.0%) for CML, 8.8% (8.5%, 9.2%) for ALL, 6.8% (6.7%, 6.9%) for NHL, 5.5% (5.3%, 5.7%) for HL and 4.5% (4.3%, 4.6%) for PCM (Fig. 2).

### Non-cancer causes of death

**Fig. 4:**
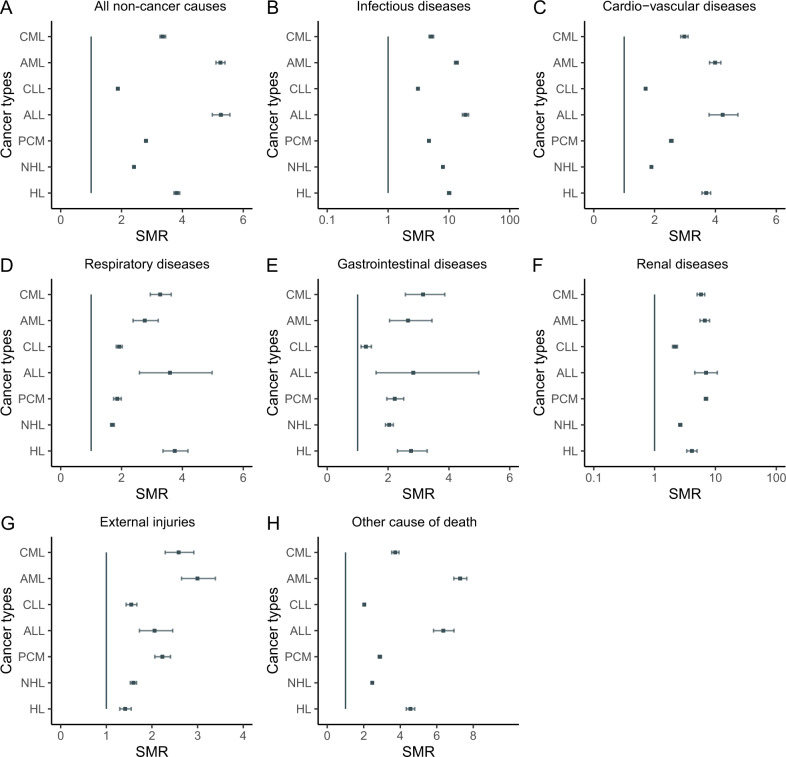
SMRs of non-cancer deaths. **A** All non-cancer deaths, **B** Infection, **C** Cardio-vascular disease, **D** Respiratory disease, **E** Gastro-intestinal disease, **F** Kidney disease, **G** External injuries, **H** Other.

Non-cancer causes were the cause of death in persons with CLL (44%), HL (38%), NHL (33%), CML (29%), PCM (24%), ALL (11%) and AML (10%; Fig. 3, Tables S2–S8). The all-cause SMR of non-cancer deaths was highest in persons with ALL (all-cause SMR = 5.25 [4.97, 5.55) followed by AML (all-cause SMR = 5.13 [4.99, 5.28) and HL (all-cause SMR = 3.80 [3.71, 3.90]; Fig. 4A). 10-year actuarial rates of non-index-cancer deaths were 25.1% (24.8%, 25.5%) for CLL, 19.7% (19.4%, 20.0%) for PCM, 19.7% (19.2%, 20.3%) for CML, 18.0% (17.9%, 18.2%) for NHL, 8.8% (8.6%, 9.1%) for HL, 8.0% (7.8%, 8.3%) for AML and 4.2% (4.0%, 4.5%) for ALL (Fig. 2).

Infections were a cause of non-cancer deaths with exceeding risks in several cancers including ALL (cause-specific SMR = 19 [17, 21), AML (cause-specific SMR = 13 [12, 14]) and HL (cause-specific SMR = 10 [9.5, 10.7]; Fig. 4, Tables S2–S8). CVD was a common non-cancer cause of death in several cancers including CLL (22%), HL (15%), NHL (14%), CML (14%) and PCM (12%; Fig. 3, Tables S2–S8). CVD was associated with the highest SMRs in ALL (cause-specific SMR = 4.22 [3.78, 4.72), AML (cause-specific SMR = 3.89 [3.71, 4.08]) and HL (cause-specific SMR = 3.68 [3.54, 3.83; Figs. 3 and 4C, Tables S2–S8). Cause-specific SMRs for respiratory deaths was high in HL (cause-specific SMR = 3.73 [3.35, 4.17]) and for kidney disease in PCM (cause-specific SMR = 6.96; [6.55, 7.41; Fig. 4).

### Cause of death per interval after diagnosis

**Fig. 5:**
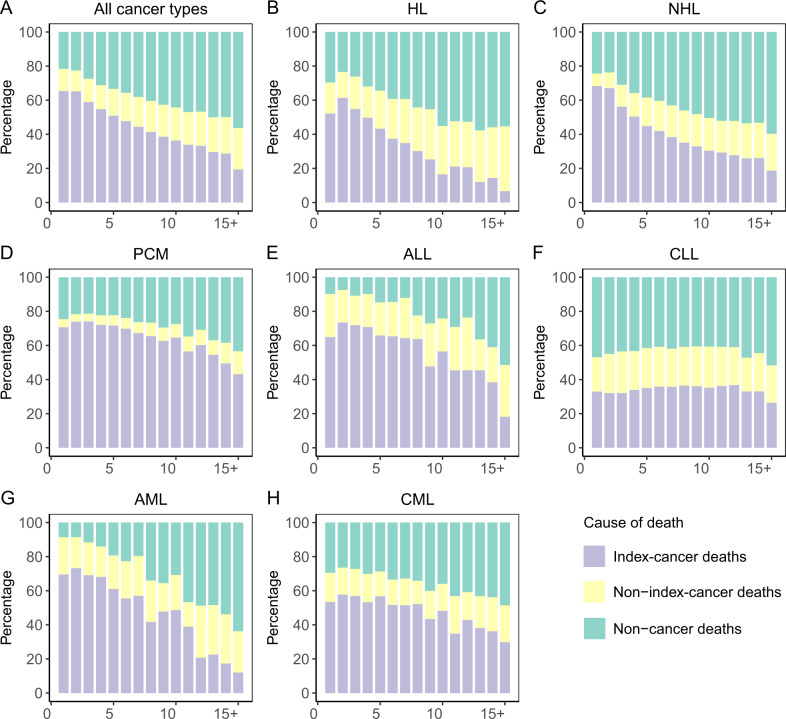
Cause of death from index cancer, new cancer or non-cancer by interval after index cancer diagnosis. **A** All haematological cancers, **B** HL, **C** NHL, **D** PCM, **E** ALL, **F** CLL, **G** AML, **H** CML.

**Fig. 6:**
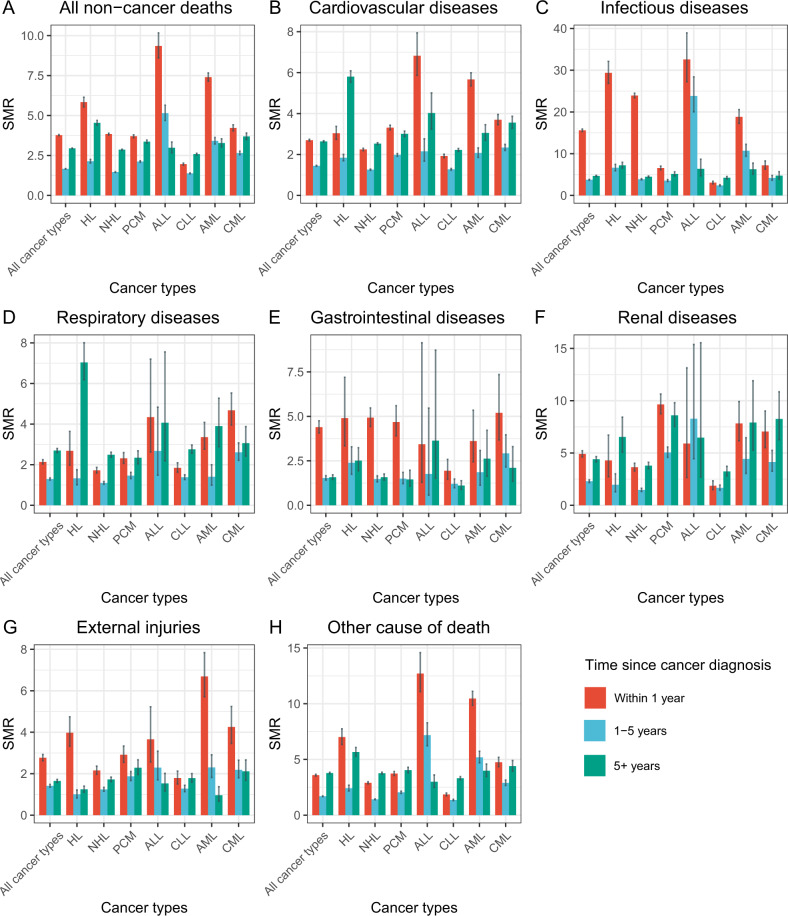
SMRs of non-cancer deaths amongst people with haematological cancer by interval after diagnosis. **A** All non-cancer deaths, **C** Infection, **B** Cardio-vascular disease, **D** Respiratory disease, **E** Gastro-intestinal disease, **F** Kidney disease, **G** External injuries, **H** Other.

The interval immediately after a haematological cancer diagnosis had the highest risk of index-cancer-specific deaths except for CLL (Fig. 5). Subsequently, the likelihood of deaths from non-cancer causes increased in long-term survivors. In CLL, non-cancer causes were the leading cause of death regardless of post-diagnosis interval (Fig. 5). Persons with cancer had higher SMRs compared with the general population throughout the follow-up interval (Fig. 6). Most cancer types had high all-cause SMRs of non-cancer deaths in the 1st year after the haematological cancer diagnosis. All-cause SMRs decreased slightly during the next 4 years and then either decreased slightly or increased thereafter. For some non-cancer causes, the cause-specific SMR was highest in the 1st year after diagnosis including infections and external injuries. For other non-cancer causes, the cause-specific SMR in the interval after 5 years after haematological cancer diagnosis was like that or less than in the 5 years following diagnosis including CVD and respiratory and kidney diseases. People with newly-diagnosed ALL and AML had the highest all-cause SMRs of non-cancer deaths in the 1^st^ year after diagnosis. We also observed extremely high cause-specific SMR for infections and external injuries in persons with newly-diagnosed HL whereas an extremely high cause-specific SMR for respiratory disease was seen in long-term HL survivors. Trends are displayed in Fig. 6.

## Discussion

We analyzed causes of death amongst 686,867 persons with haematologic cancers registered in SEER 1975-2016. Non-cancer deaths now exceed index-cancer deaths in CLL and will likely surpass index-cancer deaths soon in HL and CML. Causes of death varied by era of cancer diagnosis, cancer type, interval after diagnosis to death and other co-variates, including age and race. As cancer therapy has improved, new cancers and non-cancer causes of death are increasing in long-term cancer survivors.

We found risk of death from the index cancer, another cancer and non-cancer causes has changed for some but not all haematological cancers. As people with haematological cancer survive longer, risks of death from a new cancer and those from age-related non-cancer causes increase. There are many reasons why death from an index haematological cancer might decrease, including earlier diagnosis, better therapies, increased access to health care and others including changes in how deaths are coded and reported. Our analyses focused on characterizing cause of death amongst people with haematological cancer, not elucidating the reasons of changes which are likely complex. Such an analysis requires detailed subject-level data which are generally unavailable in population-based cancer registries such as SEER.

Our study is comprehensive but has several limitations. First, it is descriptive and retrospective. Second, cause of death may have been mis-classified because of inaccurate coding of death certificates in different intervals. This is an unlikely cause of substantial bias [25–28]. Rules for coding deaths changed during our study interval. It is also possible there might be a difference in accuracy of the cause of death in different cancers and non-cancer causes. Nevertheless, the SEER programme used standardized data collection procedures to ensure accuracy [28]. This approach has been validated [25–27, 29, 30].

## Conclusion

Risk of death from an index cancer, new cancer or non-cancer causes varied by haematological cancer type, era of diagnosis, interval after diagnosis and other co-variates including age and race. Non-cancer deaths now exceed index-cancer deaths in CLL and will become dominants soon in HL and CML. Index-cancer deaths remained dominant cause of death amongst persons with PCM, ALL and AML in the 2010s. Changes in causes of death amongst people with different haematological cancers suggest distinct strategies to improve survival, such as increased screening for new cancers or non-cancer comorbidities. Non-cancer deaths might be decreased, prevented or effectively treated, especially in persons with CLL, HL and CML. In other haematological cancers including PCM, ALL and AML, preventing death from the index cancer remains the most pressing goal.

## Supplementary information

Supplementary figures and tables

## Data Availability

Data can be accessed from the Surveillance, Epidemiology, and End Results (SEER) database at https://seer.cancer.gov/data/.
