# Supplementary material for: Changing causes of death in persons with haematological cancers 1975–2016
Source: Leukemia. 2022 May 16;36(7):1850–60. doi: 10.1038/s41375-022-01596-z (PMC9252904; doi:10.1038/s41375-022-01596-z)
Supplement: Supplementary file 1 — Supplementary figures and tables [file 41375_2022_1596_MOESM1_ESM.docx]

**Supplementary Figures and Tables**

**Figure S1** Inclusion and exclusion criteria of patients in this study.

**Table S1** Trends of deaths from the index cancer, non-index cancer or non-cancer causes among patients with haematological cancer by year of diagnosis from 1975 to 2016, related to Figure 1.

**Table S2** Cause of deaths among patients with ALL, related to Figure 3 and Figure 4.

**Table S3** Cause of deaths among patients with AML, related to Figure 3 and Figure 4.

**Table S4** Cause of deaths among patients with CLL, related to Figure 3 and Figure 4.

**Table S5** Cause of deaths among patients with CML, related to Figure 3 and Figure 4.

**Table S6** Cause of deaths among patients with HL, related to Figure 3 and Figure 4.

**Table S7** Cause of deaths among patients with PCM, related to Figure 3 and Figure 4.

**Table S8** Cause of deaths among patients with NHL, related to Figure 3 and Figure 4.


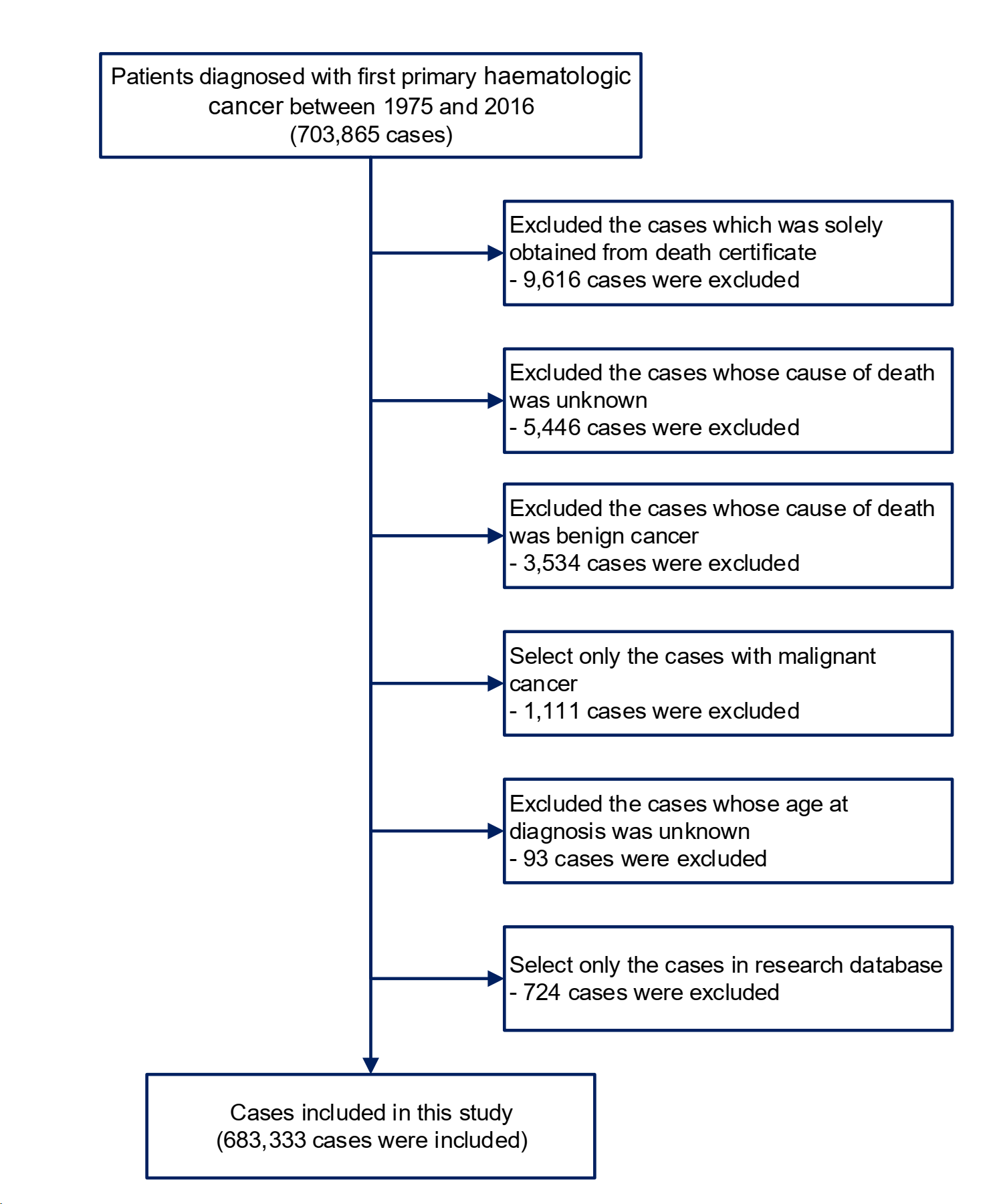


**Figure S1** Inclusion and exclusion criteria of patients in this study.

**Table S1** Trends of deaths from the index cancer, non-index cancer or non-cancer causes among patients with haematological cancer by year of diagnosis from 1975 to 2016, related to Figure 1.

|  | Trend 1^1,2^ | | |  | Trend 2^1,2^ | | |  | Overall trend ^1,2^ | |
| --- | --- | --- | --- | --- | --- | --- | --- | --- | --- | --- |
|  | Time | 5-year PC | *p* |  | Time | 5-year PC | p |  | Average 5-year PC | p |
| All cancer types |  |  |  |  |  |  |  |  |  |  |
| Index cancer | 1975-1979 to 2010-2014 | -1.02 | **0.04** |  |  |  |  |  | -1.02 | **0.04** |
| Non-index cancer | 1975-1979 to 2010-2014 | -2.05 | **0.04** |  |  |  |  |  | -2.05 | **0.04** |
| Non-cancer | 1975-1979 to 2010-2014 | 3.49 | **0.02** |  |  |  |  |  | 3.49 | **0.02** |
| HL |  |  |  |  |  |  |  |  |  |  |
| Index cancer | 1975-1979 to 2010-2014 | -5.91 | **< 0.001** |  |  |  |  |  | -5.91 | **< 0.001** |
| Non-index cancer | 1975-1979 to 2010-2014 | 5.41 | **0.001** |  |  |  |  |  | 5.41 | **0.001** |
| Non-cancer | 1975-1979 to 2010-2014 | 6.53 | **0.01** |  |  |  |  |  | 6.53 | **0.01** |
| NHL |  |  |  |  |  |  |  |  |  |  |
| Index cancer | 1975-1979 to 2010-2014 | -2.01 | **0.02** |  |  |  |  |  | -2.01 | **0.02** |
| Non-index cancer | 1975-1979 to 2010-2014 | 0.3 | 0.9 |  |  |  |  |  | 0.3 | 0.9 |
| Non-cancer | 1975-1979 to 1990-1994 | 16.86 | 0.07 |  | 1990-1994 to 2010-2014 | -2.09 | 0.5 |  | 5.62 | 0.07 |
| PCM |  |  |  |  |  |  |  |  |  |  |
| Index cancer | 1975-1979 to 2010-2014 | -1.26 | **0.001** |  |  |  |  |  | -1.26 | **0.001** |
| Non-index cancer | 1975-1979 to 2010-2014 | 3.83 | **0.04** |  |  |  |  |  | 3.83 | **0.04** |
| Non-cancer | 1975-1979 to 1995-1999 | 1.47 | 0.34 |  | 1995-1999 to 2010-2014 | 6.09 | 0.04 |  | 3.43 | **0.001** |
| ALL |  |  |  |  |  |  |  |  |  |  |
| Index cancer | 1975-1979 to 2010-2014 | -0.3 | 0.5 |  |  |  |  |  | -0.3 | 0.5 |
| Non-index cancer | 1975-1979 to 2010-2014 | -2.04 | **0.03** |  |  |  |  |  | -2.04 | **0.03** |
| Non-cancer | 1975-1979 to 2010-2014 | 6.44 | **0.01** |  |  |  |  |  | 6.44 | **0.01** |
| CLL |  |  |  |  |  |  |  |  |  |  |
| Index cancer | 1975-1979 to 2010-2014 | -4.69 | **0.004** |  |  |  |  |  | -4.69 | **0.004** |
| Non-index cancer | 1975-1979 to 2010-2014 | 2.75 | **0.03** |  |  |  |  |  | 2.75 | **0.03** |
| Non-cancer | 1975-1979 to 2010-2014 | 2.95 | **0.04** |  |  |  |  |  | 2.95 | **0.04** |
| AML |  |  |  |  |  |  |  |  |  |  |
| Index cancer | 1975-1979 to 1985-1989 | -4.89 | 0.2 |  | 1985-1989 to 2010-2014 | 4.73 | **0.01** |  | 1.9 | 0.07 |
| Non-index cancer | 1975-1979 to 1985-1989 | 12.06 | **0.046** |  | 1985-1989 to 2010-2014 | -12.69 | **< 0.001** |  | -6.24 | **< 0.001** |
| Non-cancer | 1975-1979 to 2010-2014 | -1.2 | 0.5 |  |  |  |  |  | -1.2 | 0.5 |
| CML |  |  |  |  |  |  |  |  |  |  |
| Index cancer | 1975-1979 to 2010-2014 | -5.2 | **< 0.001** |  |  |  |  |  | -5.2 | **< 0.001** |
| Non-index cancer | 1975-1979 to 2010-2014 | 4.84 | 0.08 |  |  |  |  |  | 4.84 | 0.08 |
| Non-cancer | 1975-1979 to 2010-2014 | 9.27 | **< 0.001** |  |  |  |  |  | 9.27 | **< 0.001** |

Notes:

1. 5-year PC = percentage change by 5 years, average 5-year PC = average percentage change by 5 years; The *p-value* lower than 0.05 indicate that the 5-year PC or average 5-year PC of trends were significantly different than zero.

2. Due to the limited number of points to fit the model, the maximal *joinpoints* were limited to 1.

**Table S2** Cause of deaths among patients with ALL, related to Figure 3 and Figure 4.

| Causes of death | Cancer patients | |  | General population | | SMR (95% CI) |
| --- | --- | --- | --- | --- | --- | --- |
|  | Observed No. of deaths (%) | Mortality rates in cancer patients |  | Expected No. of deaths | Mortality rates in general population |  |
| **All causes** | 11,505 (100%) | 4,913.8 |  |  |  |  |
| **Index cancer** | 7,649 (66%) | 3,258.1 |  |  |  |  |
| **Non**-**index cancer** | 2,573 (22%) | 1,109.2 |  |  |  |  |
| **Non**-**cancer cause of death** | 1,283 (11%) | 546.5 |  | 244.3 | 104.1 | 5.25 (4.97-5.55) |
| **Infectious diseases** | 285 (3%) | 121.4 |  | 15.2 | 6.5 | 18.7 (16.6-21.0) |
| Pneumonia and influenza | 68 (0.6%) | 29.0 |  | 6.3 | 2.7 | 10.8 (8.54-13.7) |
| Syphilis | 1 (0.01%) | 0.4 |  | 0.004 | 0.002 | 249.3 (35.1-1,769.8) |
| Tuberculosis | 1 (0.01%) | 0.4 |  | 0.11 | 0.05 | 9.00 (1.27-63.9) |
| Septicemia | 53 (0.5%) | 22.6 |  | 3.2 | 1.3 | 16.8 (12.8-21.9) |
| Other infections | 162 (1%) | 69.0 |  | 5.6 | 2.4 | 29.0 (24.8-33.8) |
| **Cardiovascular diseases** | 308 (3%) | 131.2 |  | 73.0 | 31.1 | 4.22 (3.78-4.72) |
| Diseases of heart | 228 (2%) | 97.1 |  | 57.3 | 24.4 | 3.98 (3.49-4.53) |
| Hypertension without heart disease | 7 (0.06%) | 3.0 |  | 1.4 | 0.6 | 5.17 (2.46-10.8) |
| Aortic aneurysm and dissection | 4 (0.03%) | 1.7 |  | 1.3 | 0.5 | 3.19 (1.20-8.49) |
| Atherosclerosis | 3 (0.03%) | 1.3 |  | 0.9 | 0.4 | 3.48 (1.12-10.8) |
| Cerebrovascular diseases | 63 (0.5%) | 26.8 |  | 11.3 | 4.8 | 5.56 (4.34-7.12) |
| Other diseases of arteries, arterioles, capillaries | 3 (0.03%) | 1.3 |  | 0.7 | 0.3 | 4.06 (1.31-12.6) |
| **Respiratory diseases** | 36 (0.3%) | 15.3 |  | 10.1 | 4.3 | 3.57 (2.58-4.96) |
| Chronic obstructive pulmonary disease and allied cond | 36 (0.3%) | 15.3 |  | 10.1 | 4.3 | 3.57 (2.58-4.96) |
| **Gastrointestinal diseases** | 12 (0.1%) | 5.1 |  | 4.3 | 1.8 | 2.82 (1.60-4.97) |
| Stomach and duodenal ulcers | 3 (0.03%) | 1.3 |  | 0.4 | 0.2 | 7.85 (2.53-24.3) |
| Chronic liver disease and cirrhosis | 9 (0.08%) | 3.8 |  | 3.9 | 1.6 | 2.34 (1.22-4.49) |
| **Renal diseases** | 21 (0.2%) | 8.9 |  | 3.0 | 1.3 | 6.97 (4.54-10.7) |
| Nephritis, nephrotic syndrome and nephrosis | 21 (0.2%) | 8.9 |  | 3.0 | 1.3 | 6.97 (4.54-10.69) |
| **External injuries** | 124 (1%) | 52.8 |  | 60.3 | 25.7 | 2.05 (1.72-2.45) |
| Accidents and adverse effects | 91 (0.8%) | 38.8 |  | 44.5 | 19.0 | 2.04 (1.66-2.51) |
| Suicide and self-inflicted injury | 21 (0.2%) | 8.9 |  | 8.3 | 3.5 | 2.54 (1.66-3.90) |
| **Other cause of death** | 497 (4%) | 211.7 |  | 78.4 | 33.4 | 6.34 (5.81-6.93) |
| Homicide and legal intervention | 12 (0.1%) | 5.1 |  | 7.6 | 3.2 | 1.59 (0.90-2.79) |
| Alzheimers | 9 (0.08%) | 3.8 |  | 2.6 | 1.1 | 3.47 (1.81-6.67) |
| Diabetes mellitus | 26 (0.2%) | 11.1 |  | 5.9 | 2.5 | 4.43 (3.01-6.50) |
| Congenital anomalies | 14 (0.1%) | 6.0 |  | 12.9 | 5.5 | 1.09 (0.64-1.84) |
| Certain conditions originating in perinatal period | 1 (0.01%) | 0.4 |  | 14.3 | 6.1 | 0.07 (0.01-0.50) |
| Complications of pregnancy, childbirth, puerperium | 4 (0.03%) | 1.7 |  | 0.1 | 0.03 | 49.5 (18.6-131.9) |
| Symptoms, signs and ill-defined conditions | 30 (0.3%) | 12.8 |  | 8.6 | 3.7 | 3.50 (2.44-5.00) |
| Other cause of death | 413 (4%) | 175.9 |  | 34.0 | 14.5 | 12.2 (11.0-13.4) |

**Table S3** Cause of deaths among patients with AML, related to Figure 3 and Figure 4.

| Causes of death | Cancer patients | |  | General population | | SMR (95% CI) |
| --- | --- | --- | --- | --- | --- | --- |
|  | Observed No. of deaths (%) | Mortality rates in cancer patients |  | Expected No. of deaths | Mortality rates in general population |  |
| **All causes** | 47,324 (100%) | 33,266.6 |  |  |  |  |
| **Index cancer** | 32,589 (68%) | 22,570.0 |  |  |  |  |
| **Non**-**index cancer** | 9,990 (21%) | 7,410.4 |  |  |  |  |
| **Non**-**cancer cause of death** | 4,745 (10%) | 3,286.2 |  | 924.4 | 640.2 | 5.13 (4.99-5.28) |
| **Infectious diseases** | 794 (2%) | 549.9 |  | 61.3 | 42.5 | 13.0 (12.1-13.9) |
| Pneumonia and influenza | 285 (0.6%) | 197.4 |  | 29.2 | 20.2 | 9.75 (8.68-11.0) |
| Syphilis | 0 (0.00%) | 0.0 |  | 0.0 | 0.0 | NA |
| Tuberculosis | 2 (0.00%) | 1.4 |  | 0.7 | 0.5 | 3.04 (0.76-12.1) |
| Septicemia | 173 (0.4%) | 119.8 |  | 14.6 | 10.1 | 11.8 (10.2-13.8) |
| Other infections | 334 (0.7%) | 231.3 |  | 16.8 | 11.6 | 19.9 (17.9-22.2) |
| **Cardiovascular diseases** | 1,742 (4%) | 1,206.4 |  | 447.8 | 310.1 | 3.89 (3.71-4.08) |
| Diseases of heart | 1,417 (2.9%) | 981.4 |  | 348.9 | 241.6 | 4.06 (3.86-4.28) |
| Hypertension without heart disease | 41 (0.09%) | 28.4 |  | 8.9 | 6.2 | 4.59 (3.38-6.23) |
| Aortic aneurysm and dissection | 12 (0.02%) | 8.3 |  | 7.9 | 5.5 | 1.51 (0.86-2.66) |
| Atherosclerosis | 10 (0.02%) | 6.9 |  | 6.1 | 4.2 | 1.64 (0.88-3.05) |
| Cerebrovascular diseases | 239 (0.5%) | 165.5 |  | 71.2 | 49.3 | 3.36 (2.96-3.81) |
| Other diseases of arteries, arterioles, capillaries | 23 (0.05%) | 15.9 |  | 4.7 | 3.2 | 4.91 (3.26-7.39) |
| **Respiratory diseases** | 173 (0.4%) | 119.8 |  | 64.2 | 44.5 | 2.69 (2.32-3.13) |
| Chronic obstructive pulmonary disease and allied cond | 173 (0.4%) | 119.8 |  | 64.2 | 44.5 | 2.69 (2.32-3.13) |
| **Gastrointestinal diseases** | 57 (0.1%) | 39.5 |  | 21.7 | 15.1 | 2.62 (2.02-3.40) |
| Stomach and duodenal ulcers | 12 (0.02%) | 8.3 |  | 2.3 | 1.6 | 5.19 (2.94-9.13) |
| Chronic liver disease and cirrhosis | 45 (0.09%) | 31.2 |  | 19.4 | 13.4 | 2.32 (1.73-3.10) |
| **Renal diseases** | 118 (0.2%) | 81.7 |  | 18.2 | 12.6 | 6.50 (5.42-7.78) |
| Nephritis, nephrotic syndrome and nephrosis | 118 (0.2%) | 81.7 |  | 18.2 | 12.6 | 6.50 (5.42-7.78) |
| **External injuries** | 252 (0.5%) | 174.5 |  | 85.0 | 58.9 | 2.96 (2.62-3.35) |
| Accidents and adverse effects | 198 (0.4%) | 137.1 |  | 57.1 | 39.5 | 3.47 (3.02-3.99) |
| Suicide and self-inflicted injury | 47 (0.1%) | 32.6 |  | 19.3 | 13.3 | 2.44 (1.83-3.25) |
| **Other cause of death** | 1,314 (3%) | 910.0 |  | 138.3 | 95.8 | 9.50 (9.00-10.0) |
| Homicide and legal intervention | 7 (0.01%) | 4.8 |  | 8.7 | 6.0 | 0.81 (0.38-1.69) |
| Alzheimers | 26 (0.05%) | 18.0 |  | 19.2 | 13.3 | 1.36 (0.92-1.99) |
| Diabetes mellitus | 95 (0.2%) | 65.8 |  | 36.0 | 24.9 | 2.64 (2.16-3.23) |
| Congenital anomalies | 48 (0.1%) | 33.2 |  | 7.0 | 4.8 | 6.88 (5.18-9.13) |
| Certain conditions originating in perinatal period | 9 (0.02%) | 6.2 |  | 9.6 | 6.7 | 0.93 (0.49-1.80) |
| Complications of pregnancy, childbirth, puerperium | 22 (0.05%) | 15.2 |  | 0.2 | 0.1 | 107.8 (71.0-163.7) |
| Symptoms, signs and ill-defined conditions | 95 (0.2%) | 65.8 |  | 15.7 | 10.9 | 6.05 (4.94-7.39) |
| Other cause of death | 1,609 (3%) | 1,114.3 |  | 226.1 | 156.6 | 7.12 (6.78-7.47) |

**Table S4** Cause of deaths among patients with CLL, related to Figure 3 and Figure 4.

| Causes of death | Cancer patients | |  | General population | | SMR (95% CI) |
| --- | --- | --- | --- | --- | --- | --- |
|  | Observed No. of deaths (%) | Mortality rates in cancer patients |  | Expected No. of deaths | Mortality rates in general population |  |
| **All causes** | 42,383 (100%) | 8,998.7 |  |  |  |  |
| **Index cancer** | 14,225 (34%) | 3,000.7 |  |  |  |  |
| **Non**-**index cancer** | 9,522 (22%) | 2,066.8 |  |  |  |  |
| **Non**-**cancer cause of death** | 18,636 (44%) | 3,931.2 |  | 9,964.8 | 2,102.0 | 1.87 (1.84-1.90) |
| **Infectious diseases** | 1,929 (5%) | 406.9 |  | 626.3 | 132.1 | 3.08 (2.95-3.22) |
| Pneumonia and influenza | 1,174 (3%) | 247.7 |  | 377.3 | 79.6 | 3.11 (2.94-3.29) |
| Syphilis | 0 (0.0%) | 0.0 |  | 0.2 | 0.0 | NA |
| Tuberculosis | 9 (0.02%) | 1.9 |  | 6.0 | 1.3 | 1.50 (0.78-2.88) |
| Septicemia | 338 (0.8%) | 71.3 |  | 153.1 | 32.3 | 2.21 (1.98-2.46) |
| Other infections | 408 (1%) | 86.1 |  | 89.5 | 18.9 | 4.56 (4.14-5.02) |
| **Cardiovascular diseases** | 9,345 (22%) | 1,971.3 |  | 5,501.4 | 1,160.5 | 1.70 (1.66-1.73) |
| Diseases of heart | 7,477 (18%) | 1,577.2 |  | 4,265.7 | 899.8 | 1.75 (1.71-1.79) |
| Hypertension without heart disease | 201 (0.5%) | 42.4 |  | 100.7 | 21.2 | 2.00 (1.74-2.29) |
| Aortic aneurysm and dissection | 113 (0.3%) | 23.8 |  | 98.0 | 20.7 | 1.15 (0.96-1.39) |
| Atherosclerosis | 153 (0.4%) | 32.3 |  | 89.2 | 18.8 | 1.71 (1.46-2.01) |
| Cerebrovascular diseases | 1,310 (3%) | 276.3 |  | 891.3 | 188.0 | 1.47 (1.39-1.55) |
| Other diseases of arteries, arterioles, capillaries | 91 (0.2%) | 19.2 |  | 56.4 | 11.9 | 1.61 (1.31-1.98) |
| **Respiratory diseases** | 1,453 (3%) | 306.5 |  | 759.1 | 160.1 | 1.91 (1.82-2.02) |
| Chronic obstructive pulmonary disease and allied cond | 1,453 (3%) | 306.5 |  | 759.1 | 160.1 | 1.91 (1.82-2.02) |
| **Gastrointestinal diseases** | 215 (0.5%) | 45.4 |  | 169.7 | 35.8 | 1.27 (1.11-1.45) |
| Stomach and duodenal ulcers | 47 (0.1%) | 9.9 |  | 28.2 | 6.0 | 1.66 (1.25-2.21) |
| Chronic liver disease and cirrhosis | 168 (0.4%) | 35.4 |  | 141.5 | 29.8 | 1.19 (1.02-1.38) |
| **Renal diseases** | 441 (1%) | 93.0 |  | 204.6 | 43.2 | 2.16 (1.96-2.37) |
| Nephritis, nephrotic syndrome and nephrosis | 441 (1%) | 93.0 |  | 204.6 | 43.2 | 2.16 (1.96-2.37) |
| **External injuries** | 665 (2%) | 140.3 |  | 431.2 | 91.0 | 1.54 (1.43-1.66) |
| Accidents and adverse effects | 543 (1%) | 114.5 |  | 321.1 | 67.7 | 1.69 (1.55-1.84) |
| Suicide and self-inflicted injury | 106 (0.2%) | 22.4 |  | 91.4 | 19.3 | 1.16 (0.96-1.40) |
| **Other cause of death** | 3,224 (8%) | 680.1 |  | 1,490.8 | 314.5 | 2.16 (2.09-2.24) |
| Homicide and legal intervention | 16 (0.04%) | 3.4 |  | 18.7 | 3.9 | 0.86 (0.53-1.40) |
| Alzheimers | 536 (1%) | 113.1 |  | 277.3 | 58.5 | 1.93 (1.78-2.10) |
| Diabetes mellitus | 594 (1%) | 125.3 |  | 362.3 | 76.4 | 1.64 (1.51-1.78) |
| Congenital anomalies | 20 (0.05%) | 4.2 |  | 12.9 | 2.7 | 1.55 (1.00-2.40) |
| Certain conditions originating in perinatal period | 2 (0.005%) | 0.4 |  | 0.0 | 0.0 | 262.4 (65.6-1,049.3) |
| Complications of pregnancy, childbirth, puerperium | 1 (0.002%) | 0.2 |  | 0.1 | 0.0 | 16.4 (2.32-116.7) |
| Symptoms, signs and ill-defined conditions | 211 (0.5%) | 44.5 |  | 129.1 | 27.2 | 1.63 (1.43-1.87) |
| Other cause of death | 4,588 (11%) | 967.8 |  | 2,272.6 | 479.4 | 2.02 (1.96-2.08) |

**Table S5** Cause of deaths among patients with CML, related to Figure 3 and Figure 4.

| Causes of death | Cancer patients | |  | General population | | SMR (95% CI) |
| --- | --- | --- | --- | --- | --- | --- |
|  | Observed No. of deaths (%) | Mortality rates in cancer patients |  | Expected No. of deaths | Mortality rates in general population |  |
| **All causes** | 17,343 (100%) | 12,912.2 |  |  |  |  |
| **Index cancer** | 8,940 (53%) | 6,656.0 |  |  |  |  |
| **Non**-**index cancer** | 2,750 (16%) | 2,493.4 |  |  |  |  |
| **Non**-**cancer cause of death** | 5,054 (30%) | 3,762.8 |  | 1,547.5 | 1,152.1 | 3.27 (3.18-3.36) |
| **Infectious diseases** | 523 (3%) | 389.4 |  | 104.6 | 77.9 | 5.00 (4.59-5.45) |
| Pneumonia and influenza | 242 (1%) | 180.2 |  | 56.2 | 41.9 | 4.30 (3.79-4.88) |
| Syphilis | 0 (0.0%) | 0.0 |  | 0.0 | 0.0 | NA |
| Tuberculosis | 5 (0.03%) | 3.7 |  | 1.0 | 0.8 | 4.86 (2.02-11.7) |
| Septicemia | 102 (0.6%) | 75.9 |  | 24.6 | 18.3 | 4.15 (3.42-5.04) |
| Other infections | 174 (1.0%) | 129.5 |  | 22.7 | 16.9 | 7.67 (6.61-8.90) |
| **Cardiovascular diseases** | 2,325 (14%) | 1,731.0 |  | 803.8 | 598.4 | 2.89 (2.78-3.01) |
| Diseases of heart | 1,842 (11%) | 1,371.4 |  | 621.5 | 462.7 | 2.96 (2.83-3.10) |
| Hypertension without heart disease | 65 (0.4%) | 48.4 |  | 16.3 | 12.2 | 3.98 (3.12-5.07) |
| Aortic aneurysm and dissection | 28 (0.2%) | 20.8 |  | 13.7 | 10.2 | 2.04 (1.41-2.96) |
| Atherosclerosis | 44 (0.3%) | 32.8 |  | 12.3 | 9.2 | 3.56 (2.65-4.79) |
| Cerebrovascular diseases | 302 (2%) | 224.8 |  | 131.7 | 98.0 | 2.29 (2.05-2.57) |
| Other diseases of arteries, arterioles, capillaries | 44 (0.3%) | 32.8 |  | 8.2 | 6.1 | 5.34 (3.97-7.17) |
| **Respiratory diseases** | 348 (2%) | 259.1 |  | 109.5 | 81.5 | 3.18 (2.86-3.53) |
| Chronic obstructive pulmonary disease and allied cond | 348 (2%) | 259.1 |  | 109.5 | 81.5 | 3.18 (2.86-3.53) |
| **Gastrointestinal diseases** | 92 (0.5%) | 68.5 |  | 29.7 | 22.1 | 3.10 (2.53-3.80) |
| Stomach and duodenal ulcers | 29 (0.2%) | 21.6 |  | 4.1 | 3.0 | 7.11 (4.94-10.2) |
| Chronic liver disease and cirrhosis | 63 (0.4%) | 46.9 |  | 25.6 | 19.1 | 2.46 (1.92-3.15) |
| **Renal diseases** | 181 (1%) | 134.8 |  | 32.2 | 24.0 | 5.61 (4.85-6.49) |
| Nephritis, nephrotic syndrome and nephrosis | 181 (1%) | 134.8 |  | 32.2 | 24.0 | 5.61 (4.85-6.49) |
| **External injuries** | 261 (2%) | 194.3 |  | 102.5 | 76.3 | 2.55 (2.26-2.88) |
| Accidents and adverse effects | 227 (1%) | 169.0 |  | 71.0 | 52.9 | 3.20 (2.81-3.64) |
| Suicide and self-inflicted injury | 31 (0.2%) | 23.1 |  | 22.4 | 16.7 | 1.38 (0.97-1.97) |
| **Other cause of death** | 999 (8%) | 743.8 |  | 237.8 | 177.0 | 4.20 (3.95-4.47) |
| Homicide and legal intervention | 3 (0.02%) | 2.2 |  | 9.1 | 6.8 | 0.33 (0.11-1.03) |
| Alzheimers | 59 (0.3%) | 43.9 |  | 41.5 | 30.9 | 1.42 (1.10-1.84) |
| Diabetes mellitus | 160 (0.9%) | 119.1 |  | 57.9 | 43.1 | 2.76 (2.37-3.23) |
| Congenital anomalies | 30 (0.2%) | 22.3 |  | 3.6 | 2.7 | 8.29 (5.80-11.9) |
| Certain conditions originating in perinatal period | 1 (0.01%) | 0.7 |  | 2.0 | 1.5 | 0.49 (0.07-3.50) |
| Complications of pregnancy, childbirth, puerperium | 5 (0.03%) | 3.7 |  | 0.1 | 0.1 | 34.0 (14.1-81.6) |
| Symptoms, signs and ill-defined conditions | 70 (0.4%) | 52.1 |  | 22.1 | 16.5 | 3.17 (2.51-4.00) |
| Other cause of death | 1,324 (8%) | 985.7 |  | 365.1 | 271.9 | 3.63 (3.44-3.83) |

**Table S6** Cause of deaths among patients with HL, related to Figure 3 and Figure 4.

| Causes of death | Cancer patients | |  | General population | | SMR (95% CI) |
| --- | --- | --- | --- | --- | --- | --- |
|  | Observed No. of deaths (%) | Mortality rates in cancer patients |  | Expected No. of deaths | Mortality rates in general population |  |
| **All causes** | 16,486 (100%) | 3,011.5 |  |  |  |  |
| **Index cancer** | 6,344 (39%) | 1,158.9 |  |  |  |  |
| **Non**-**index cancer** | 3,886 (24%) | 723.2 |  |  |  |  |
| **Non**-**cancer cause of death** | 6,183 (38%) | 1,129.4 |  | 1,624.8 | 296.8 | 3.81 (3.71-3.90) |
| **Infectious diseases** | 1,161 (7%) | 212.1 |  | 115.4 | 21.1 | 10.1 (9.50-10.7) |
| Pneumonia and influenza | 270 (2%) | 49.3 |  | 41.7 | 7.6 | 6.47 (5.74-7.29) |
| Syphilis | 0 (0%) | 0.0 |  | 0.0 | 0.0 | NA |
| Tuberculosis | 3 (0.02%) | 0.5 |  | 1.4 | 0.3 | 2.15 (0.69-6.66) |
| Septicemia | 147 (0.9%) | 26.9 |  | 20.4 | 3.7 | 7.20 (6.13-8.47) |
| Other infections | 741 (5%) | 135.4 |  | 51.7 | 9.4 | 14.3 (13.3-15.4) |
| **Cardiovascular diseases** | 2,495 (15%) | 455.8 |  | 677.5 | 123.8 | 3.68 (3.54-3.83) |
| Diseases of heart | 2,126 (13%) | 388.4 |  | 537.2 | 98.1 | 3.96 (3.79-4.13) |
| Hypertension without heart disease | 46 (0.3%) | 8.4 |  | 11.6 | 2.1 | 3.98 (2.98-5.31) |
| Aortic aneurysm and dissection | 24 (0.1%) | 4.4 |  | 12.3 | 2.3 | 1.94 (1.30-2.90) |
| Atherosclerosis | 19 (0.1%) | 3.5 |  | 7.8 | 1.4 | 2.44 (1.56-3.83) |
| Cerebrovascular diseases | 258 (2%) | 47.1 |  | 101.4 | 18.5 | 2.55 (2.25-2.88) |
| Other diseases of arteries, arterioles, capillaries | 22 (0.1%) | 4.0 |  | 7.0 | 1.3 | 3.13 (2.06-4.76) |
| **Respiratory diseases** | 321 (2%) | 58.6 |  | 86.0 | 15.7 | 3.73 (3.35-4.17) |
| Chronic obstructive pulmonary disease and allied cond | 321 (2%) | 58.6 |  | 86.0 | 15.7 | 3.73 (3.35-4.17) |
| **Gastrointestinal diseases** | 123 (0.7%) | 22.5 |  | 44.8 | 8.2 | 2.74 (2.30-3.27) |
| Stomach and duodenal ulcers | 20 (0.1%) | 3.7 |  | 3.9 | 0.7 | 5.17 (3.34-8.02) |
| Chronic liver disease and cirrhosis | 103 (0.6%) | 18.8 |  | 40.9 | 7.5 | 2.52 (2.07-3.05) |
| **Renal diseases** | 99 (0.6%) | 18.1 |  | 24.2 | 4.4 | 4.09 (3.35-4.97) |
| Nephritis, nephrotic syndrome and nephrosis | 99 (0.6%) | 18.1 |  | 24.2 | 4.4 | 4.09 (3.35-4.97) |
| **External injuries** | 492 (3%) | 89.9 |  | 348.5 | 63.7 | 1.41 (1.29-1.54) |
| Accidents and adverse effects | 357 (2%) | 65.2 |  | 217.6 | 39.8 | 1.64 (1.48-1.82) |
| Suicide and self-inflicted injury | 113 (0.7%) | 20.6 |  | 77.5 | 14.2 | 1.46 (1.21-1.75) |
| **Other cause of death** | 1,492 (9%) | 272.5 |  | 328.3 | 60.0 | 4.54 (4.32-4.78) |
| Homicide and legal intervention | 22 (0.1%) | 4.0 |  | 53.4 | 9.8 | 0.41 (0.27-0.63) |
| Alzheimers | 45 (0.3%) | 8.2 |  | 20.7 | 3.8 | 2.18 (1.62-2.91) |
| Diabetes mellitus | 141 (0.9%) | 25.8 |  | 53.7 | 9.8 | 2.63 (2.23-3.10) |
| Congenital anomalies | 21 (0.1%) | 3.8 |  | 7.5 | 1.4 | 2.79 (1.82-4.28) |
| Certain conditions originating in perinatal period | 0 (0.0%) | 0.0 |  | 0.0 | 0.0 | NA |
| Complications of pregnancy, childbirth, puerperium | 4 (0.02%) | 0.7 |  | 1.2 | 0.2 | 3.25 (1.22-8.66) |
| Symptoms, signs and ill-defined conditions | 103 (0.6%) | 18.8 |  | 28.8 | 5.3 | 3.58 (2.95-4.34) |
| Other cause of death | 1,178 (7%) | 215.2 |  | 216.2 | 39.5 | 5.45 (5.15-5.77) |

**Table S7** Cause of deaths among patients with PCM, related to Figure 3 and Figure 4.

| Causes of death | Cancer patients | |  | General population | | SMR (95% CI) |
| --- | --- | --- | --- | --- | --- | --- |
|  | Observed No. of deaths (%) | Mortality rates in cancer patients |  | Expected No. of deaths | Mortality rates in general population |  |
| **All causes** | 71,934 (100%) | 20,543.4 |  |  |  |  |
| **Index cancer** | 50,520 (70%) | 14,427.8 |  |  |  |  |
| **Non**-**index cancer** | 3,892 (5%) | 1,180.6 |  |  |  |  |
| **Non**-**cancer cause of death** | 17,280 (24%) | 4,934.9 |  | 6,187.3 | 1,767.0 | 2.79 (2.75-2.83) |
| **Infectious diseases** | 1,920 (3%) | 548.3 |  | 410.5 | 117.2 | 4.68 (4.47-4.89) |
| Pneumonia and influenza | 992 (1%) | 283.3 |  | 221.3 | 63.2 | 4.48 (4.21-4.77) |
| Syphilis | 0 (0.0%) | 0.0 |  | 0.2 | 0.0 | NA |
| Tuberculosis | 11 (0.0%) | 3.1 |  | 4.7 | 1.3 | 2.37 (1.31-4.27) |
| Septicemia | 452 (0.6%) | 129.1 |  | 109.6 | 31.3 | 4.12 (3.76-4.52) |
| Other infections | 465 (0.6%) | 132.8 |  | 74.6 | 21.3 | 6.23 (5.69-6.83) |
| **Cardiovascular diseases** | 8,414 (12%) | 2,402.9 |  | 3,313.0 | 946.2 | 2.54 (2.49-2.59) |
| Diseases of heart | 6,789 (9%) | 1,938.8 |  | 2,552.4 | 728.9 | 2.66 (2.60-2.72) |
| Hypertension without heart disease | 347 (0.5%) | 99.1 |  | 74.2 | 21.2 | 4.68 (4.21-5.19) |
| Aortic aneurysm and dissection | 66 (0.1%) | 18.8 |  | 55.5 | 15.9 | 1.19 (0.93-1.51) |
| Atherosclerosis | 116 (0.2%) | 33.1 |  | 47.4 | 13.5 | 2.45 (2.04-2.94) |
| Cerebrovascular diseases | 1,025 (1%) | 292.7 |  | 548.3 | 156.6 | 1.87 (1.76-1.99) |
| Other diseases of arteries, arterioles, capillaries | 71 (0.1%) | 20.3 |  | 35.2 | 10.1 | 2.02 (1.60-2.55) |
| **Respiratory diseases** | 840 (1%) | 239.9 |  | 453.2 | 129.4 | 1.85 (1.73-1.98) |
| Chronic obstructive pulmonary disease and allied cond | 840 (1%) | 239.9 |  | 453.2 | 129.4 | 1.85 (1.73-1.98) |
| **Gastrointestinal diseases** | 245 (0.3%) | 70.0 |  | 110.7 | 31.6 | 2.21 (1.95-2.51) |
| Stomach and duodenal ulcers | 63 (0.1%) | 18.0 |  | 16.5 | 4.7 | 3.83 (2.99-4.90) |
| Chronic liver disease and cirrhosis | 182 (0.3%) | 52.0 |  | 94.3 | 26.9 | 1.93 (1.67-2.23) |
| **Renal diseases** | 1,011 (1%) | 288.7 |  | 145.2 | 41.5 | 6.96 (6.55-7.41) |
| Nephritis, nephrotic syndrome and nephrosis | 1,011 (1%) | 288.7 |  | 145.2 | 41.5 | 6.96 (6.55-7.41) |
| **External injuries** | 648 (0.9%) | 185.1 |  | 291.8 | 83.3 | 2.22 (2.06-2.40) |
| Accidents and adverse effects | 495 (0.7%) | 141.4 |  | 215.5 | 61.5 | 2.30 (2.10-2.51) |
| Suicide and self-inflicted injury | 134 (0.2%) | 38.3 |  | 57.7 | 16.5 | 2.32 (1.96-2.75) |
| **Other cause of death** | 4,202 (6%) | 1,200.0 |  | 1,462.9 | 417.8 | 2.87 (2.79-2.96) |
| Homicide and legal intervention | 19 (0.0%) | 5.4 |  | 18.6 | 5.3 | 1.02 (0.65-1.61) |
| Alzheimers | 228 (0.3%) | 65.1 |  | 160.3 | 45.8 | 1.42 (1.25-1.62) |
| Diabetes mellitus | 586 (0.8%) | 167.4 |  | 259.9 | 74.2 | 2.25 (2.08-2.44) |
| Congenital anomalies | 41 (0.1%) | 11.7 |  | 8.5 | 2.4 | 4.81 (3.54-6.54) |
| Certain conditions originating in perinatal period | 0 (0.0%) | 0.0 |  | 0.0 | 0.0 | NA |
| Complications of pregnancy, childbirth, puerperium | 5 (0.0%) | 1.4 |  | 0.1 | 0.0 | 34.5 (14.4-83.0) |
| Symptoms, signs and ill-defined conditions | 195 (0.3%) | 55.7 |  | 82.1 | 23.4 | 2.38 (2.06-2.73) |
| Other cause of death | 3,147 (4%) | 898.7 |  | 951.9 | 271.8 | 3.31 (3.19-3.42) |

**Table S8** Cause of deaths among patients with NHL, related to Figure 3 and Figure 4.

| Causes of death | Cancer patients | |  | General population | | SMR (95% CI) |
| --- | --- | --- | --- | --- | --- | --- |
|  | Observed No. of deaths (%) | Mortality rates in cancer patients |  | Expected No. of deaths | Mortality rates in general population |  |
| **All causes** | 171,236 (100%) | 9,586.4 |  |  |  |  |
| **Index cancer** | 93,070 (55%) | 5,210.4 |  |  |  |  |
| **Non**-**index cancer** | 20,740 (12%) | 1,225.8 |  |  |  |  |
| **Non**-**cancer cause of death** | 56,270 (33%) | 3,150.2 |  | 23,465.1 | 1,313.7 | 2.40 (2.38-2.42) |
| **Infectious diseases** | 12,051 (7%) | 674.7 |  | 1,521.0 | 85.2 | 7.92 (7.78-8.07) |
| Pneumonia and influenza | 2,501 (2%) | 140.0 |  | 832.6 | 46.6 | 3.00 (2.89-3.12) |
| Syphilis | 0 (0%) | 0.0 |  | 0.4 | 0.0 | NA |
| Tuberculosis | 37 (0.02%) | 2.1 |  | 15.1 | 0.8 | 2.46 (1.78-3.39) |
| Septicemia | 1,142 (0.7%) | 63.9 |  | 376.2 | 21.1 | 3.04 (2.86-3.22) |
| Other infections | 8,371 (5%) | 468.6 |  | 296.3 | 16.6 | 28.3 (27.7-28.9) |
| **Cardiovascular diseases** | 23,286 (14%) | 1,303.6 |  | 12,339.4 | 690.8 | 1.89 (1.86-1.91) |
| Diseases of heart | 18,385 (11%) | 1,029.3 |  | 9,562.5 | 535.3 | 1.92 (1.90-1.95) |
| Hypertension without heart disease | 608 (0.4%) | 34.0 |  | 243.2 | 13.6 | 2.50 (2.31-2.71) |
| Aortic aneurysm and dissection | 278 (0.2%) | 15.6 |  | 219.3 | 12.3 | 1.27 (1.13-1.43) |
| Atherosclerosis | 326 (0.2%) | 18.3 |  | 172.9 | 9.7 | 1.89 (1.69-2.10) |
| Cerebrovascular diseases | 3,441 (2.0%) | 192.6 |  | 2,011.5 | 112.6 | 1.71 (1.65-1.77) |
| Other diseases of arteries, arterioles, capillaries | 248 (0.1%) | 13.9 |  | 129.7 | 7.3 | 1.91 (1.69-2.17) |
| **Respiratory diseases** | 3,059 (2%) | 171.3 |  | 1,808.8 | 101.3 | 1.69 (1.63-1.75) |
| Chronic obstructive pulmonary disease and allied cond | 3,059 (2%) | 171.3 |  | 1,808.8 | 101.3 | 1.69 (1.63-1.75) |
| **Gastrointestinal diseases** | 935 (0.5%) | 52.3 |  | 460.3 | 25.8 | 2.03 (1.91-2.17) |
| Stomach and duodenal ulcers | 177 (0.1%) | 9.9 |  | 63.6 | 3.6 | 2.78 (2.40-3.23) |
| Chronic liver disease and cirrhosis | 758 (0.4%) | 42.4 |  | 396.6 | 22.2 | 1.91 (1.78-2.05) |
| **Renal diseases** | 1,275 (0.7%) | 71.4 |  | 484.9 | 27.1 | 2.63 (2.49-2.78) |
| Nephritis, nephrotic syndrome and nephrosis | 1,275 (0.7%) | 71.4 |  | 484.9 | 27.1 | 2.63 (2.49-2.78) |
| **External injuries** | 2,110 (1%) | 118.1 |  | 1,330.3 | 74.5 | 1.59 (1.52-1.66) |
| Accidents and adverse effects | 1,575 (0.9%) | 88.2 |  | 944.8 | 52.9 | 1.67 (1.59-1.75) |
| Suicide and self-inflicted injury | 480 (0.3%) | 26.9 |  | 292.0 | 16.3 | 1.64 (1.50-1.80) |
| **Other cause of death** | 13,554 (8%) | 758.8 |  | 5,520.3 | 309.0 | 2.46 (2.41-2.50) |
| Homicide and legal intervention | 55 (0.03%) | 3.1 |  | 93.4 | 5.2 | 0.59 (0.45-0.77) |
| Alzheimers | 1,369 (0.8%) | 76.6 |  | 642.6 | 36.0 | 2.13 (2.02-2.25) |
| Diabetes mellitus | 1,412 (0.8%) | 79.0 |  | 916.7 | 51.3 | 1.54 (1.46-1.62) |
| Congenital anomalies | 98 (0.1%) | 5.5 |  | 39.8 | 2.2 | 2.46 (2.02-3.00) |
| Certain conditions originating in perinatal period | 1 (0.00%) | 0.1 |  | 1.0 | 0.1 | 1.04 (0.15-7.35) |
| Complications of pregnancy, childbirth, puerperium | 11 (0.01%) | 0.6 |  | 1.1 | 0.1 | 9.81 (5.43-17.7) |
| Symptoms, signs and ill-defined conditions | 742 (0.4%) | 41.5 |  | 311.4 | 17.4 | 2.38 (2.22-2.56) |
| Other cause of death | 9,921 (6%) | 555.4 |  | 3,607.0 | 201.9 | 2.75 (2.70-2.81) |
